# P-127. Timing and Use of Adjunctive Steroids in Adults with Bacterial Meningitis in the United States

**DOI:** 10.1093/ofid/ofaf695.354

**Published:** 2026-01-11

**Authors:** Neha Muraly, Caroline I Reckart, Rodrigo Hasbun, Denisse Ramirez

**Affiliations:** McGovern Medical School at UTHealth Houston, Houston, TX; McGovern Medical School at the University of Texas Health Sciences Center at Houston, Houston, TX; UT Health Mc Govern Medical School, Houston, Texas; McGovern Medical School, UTHealth Science Center, Houston, TX, Houston, Texas

## Abstract

**Background:**

Adjunctive steroids decrease mortality in adults with bacterial meningitis. Steroids given within 20 minutes, 4 hours and 12 hours after the first dose of antibiotics are advocated by the Infectious Diseases Society of America (IDSA), European, and United Kingdom (UK) guidelines, respectively. Compliance with these guidelines in the United States (US) is unknown.

**Figure:**
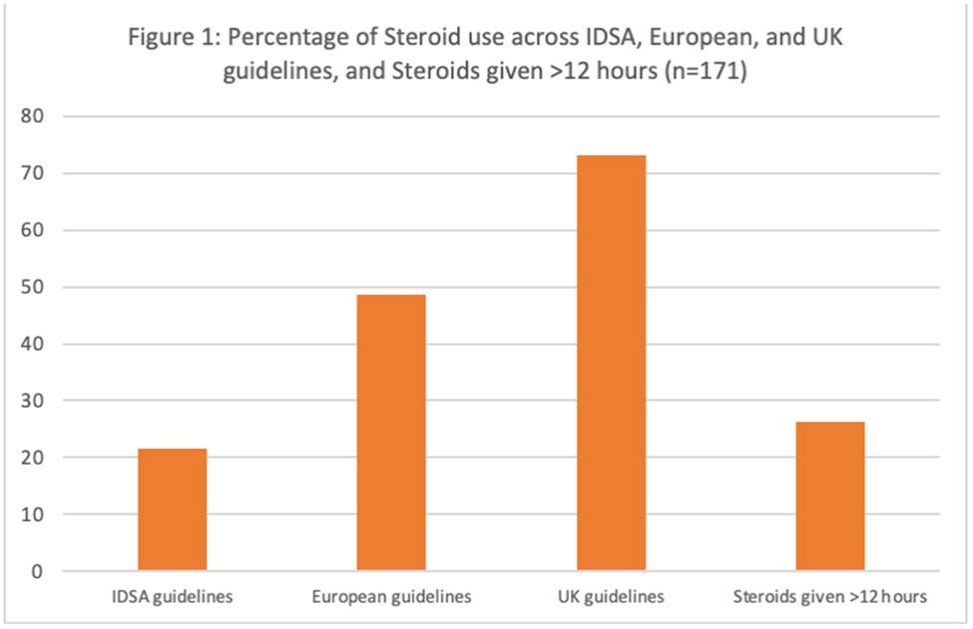

**Methods:**

Retrospective observational study of 230 adults with community-acquired bacterial meningitis at 16 hospitals in Houston, Texas from December 2004-January 2023.

**Results:**

Among the 230 patients with bacterial meningitis, a total of 171/230 (74%) got adjunctive steroids. Steroids were more likely used in patients with comorbidities, those with pneumococcal etiology, positive Gram stain, a higher CSF protein and with lower CSF glucose (P< 0.05). There was no association between the use of steroids and history of immunosuppression, fever, headache, stiff neck, Glasgow coma scale, seizures, focal neurological exam or serum WBC counts (P >0.05). Out of the 171 patients who were given steroids, 37 (22%) received steroids within 20 mins, 83 (48%) within 4 hours, and 125 (73%) within 12 hours per IDSA, European, and UK guidelines, respectively. Use of steroids in this study by any timeline was not associated with improved clinical outcomes.

**Conclusion:**

Timing and use of adjunctive steroids in adults with bacterial meningitis remain suboptimal in the US and could account for the lack of impact on clinical outcome.

**Disclosures:**

Rodrigo Hasbun, MD MPH FIDSA, Biomeriaux: Grant/Research Support|Biomeriaux: research funding and personal fees to help with Monte Carlo simulation studies evaluating impact of cost on adults and children

